# Hematopoietic Stem Cell Niche During Homeostasis, Malignancy, and Bone Marrow Transplantation

**DOI:** 10.3389/fcell.2021.621214

**Published:** 2021-01-22

**Authors:** Yan Man, Xiangmei Yao, Tonghua Yang, Yajie Wang

**Affiliations:** ^1^Department of Hematology, National Key Clinical Specialty of Hematology, Yunnan Blood Disease Clinical Medical Center, Yunnan Blood Disease Hospital, The First People’s Hospital of Yunnan Province, Kunming, China; ^2^Kunming University of Science and Technology, Kunming, China

**Keywords:** hematopoietic stem cells, hematopoietic stem cell niche, niche cells, hematologic malignancy, hematopoietic stem cell transplantation

## Abstract

Self-renewal and multidirectional differentiation of hematopoietic stem cells (HSCs) are strictly regulated by numerous cellular components and cytokines in the bone marrow (BM) microenvironment. Several cell types that regulate HSC niche have been identified, including both non-hematopoietic cells and HSC-derived cells. Specific changes in the niche composition can result in hematological malignancies. Furthermore, processes such as homing, proliferation, and differentiation of HSCs are strongly controlled by the BM niche and have been reported to be related to the success of hematopoietic stem cell transplantation (HSCT). Single-cell sequencing and *in vivo* imaging are powerful techniques to study BM microenvironment in hematological malignancies and after HSCT. In this review, we discuss how different components of the BM niche, particularly non-hematopoietic and hematopoietic cells, regulate normal hematopoiesis, and changes in the BM niche in leukemia and after HSCT. We believe that this comprehensive review will provide clues for further research on improving HSCT efficiency and exploring potential therapeutic targets for leukemia.

## Introduction

**FIGURE 1:**
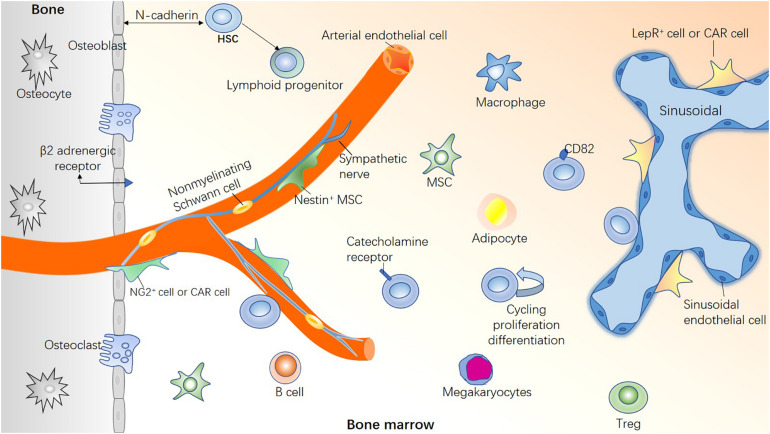
Healthy bone marrow microenvironment. If HSCs are compared to “seed,” the BM microenvironment is the “soil” for HSC survival. A healthy BM microenvironment is crucial for regulating the functional characteristics of HSCs, such as proliferation and differentiation, homing and colonization, migration and apoptosis, and maintaining steady hematopoiesis. HSCs are highly heterogeneous, with different types of HSCs located in different niches. Bone marrow microenvironment is composed of a variety of hematopoietic cells and non-hematopoietic cells, in addition, blood vessels and nerve fibers play a pivotal role.

The concept of hematopoietic stem cell (HSC) niche was first proposed by Schofield (1978), who proposed that a physical niche of stem cells exists in the bone marrow (BM). The niche consists of a variety of cells that make the microenvironment for the maintenance of stem cells. Based on his theory, advances in imaging techniques, single-cell sequencing, and molecular biology have resulted in a better understanding of HSC heterogeneity (Tikhonova et al., 2019; Christodoulou et al., 2020). As an important exogenous regulation, the BM microenvironment regulates the functional characteristics of HSCs, such as self-renewal (S), maturation (M), apoptosis (A), resting (R), and trafficking (T), together known as “SMART” properties (Cheng, 2008). The blood vessels (Chanavaz, 1995) and nerves (Maryanovich et al., 2018a) entering and exiting the BM cavity form a HSC regulatory network with non-hematopoietic cells and HSC-derived cells via mutual contact or signal transduction (Figure 1). Therefore, the loss of specific niche factors may have harmful effects on other niches.

Leukemic stem cells (LSCs) are responsible for drug resistance and relapse of leukemia (Felipe Rico et al., 2013). Leukemia cells can gradually modify the normal hematopoietic microenvironment into a leukemic microenvironment, thus contributing to disease progression (Duarte et al., 2018; Mendez-Ferrer et al., 2020). In a leukemic niche, the proliferation of normal hematopoietic stem and progenitor cells (HSPCs) is inhibited and most HSCs enter into the quiescent stage, whereas hematopoietic progenitor cells (HPCs) overproliferate and are gradually exhausted (Hu et al., 2009). For instance, Cheng et al. (2015) demonstrated that Egr3, a transcription factor, can maintain HSCs in the G0 phase, thus blocking HSC differentiation. Surprisingly, the inhibitory effect on normal hematopoiesis in leukemia is reversible (Cheng et al., 2015), implying that niche factors govern the malignant characteristics.

Hematopoietic stem cell transplantation (HSCT) is one of the most effective methods for treating hematologic malignancy. However, the occurrence of several complications, such as poor graft function (PGF), graft-vs.-host disease (GVHD), and relapse are roadblocks in improving HSCT efficiency. Moreover, total body irradiation (TBI) and chemotherapy before HSCT can damage recipient’s BM microenvironment, subsequently exerting a negative “bystander effect” on transplanted donor HSCs (Shen et al., 2012). Therefore, repairing the BM hematopoietic microenvironment would benefit leukemia treatment and prevent related complications. A recent study based on single-cell sequencing reported that transplanted HSCs decreased gradually within 1 week in myeloablated recipients, and the potential to differentiate into myeloid and erythroid lineages enhanced in some of the remaining HSCs (Dong et al., 2020). Erythropoietin (EPO) and granulocyte colony-stimulating factor (G-CSF) are key hematopoietic factors involved in erythroid and myeloid differentiation, respectively. The data showed that the concentration of EPO and G-CSF increased significantly in the microenvironment after irradiation, which could lead to the rapid differentiation of cells into these lineages (Dong et al., 2020). An in-depth study of changes in the BM microenvironment before and after HSCT is required to explore potential therapeutic targets for leukemia.

## Regulation by Non-Hematopoietic Cells

### Osteolineage Cells

Osteolineage cells are the earliest discovered niche cells that regulate HSPC (Taichman et al., 1996; Nilsson et al., 2001). Morrison (Ding and Morrison, 2013) reported that the deletion of CXCL12 from osteoblasts significantly reduced the number of early lymphoid progenitor cells, with no significant effect on HSCs and myeloid progenitor cells. The function of N-cadherin, which regulates HSCs by mediating the adhesion of homologous molecules between osteoblasts and HSCs, is also controversial (Hosokawa et al., 2010; Bromberg et al., 2012). Osteoblasts express a variety of hematopoietic regulatory molecules, including thrombopoietin (TPO) (Qian et al., 2007), angiopoietin 1 (ANGPT1) (Arai et al., 2004) and osteopontin, which negatively regulate stem cell pool size (Nilsson et al., 2005). Altogether, these studies suggest that osteoblasts do not directly regulate HSCs, at least not via CXCL12 or stem cell factor (SCF). However, osteolineage cells are speculated to support the maintenance of early lymphoid progenitors.

A previous study reported that leukemia cells initially homed and localized on the surface of osteoblasts in the epiphysial region, the most active site for the proliferation of leukemia cells (Ninomiya et al., 2007). Scadden and group (Raaijmakers et al., 2010) reported that osteoblastic progenitor cells could not differentiate into osteoblasts when Dicer1 was deleted in the mouse. Moreover, the integrity of the hematopoietic function was destroyed, eventually leading to myelodysplastic syndrome (MDS) and acute myeloid leukemia (AML). Activating mutations in β-catenin in osteoblasts can stimulate the expression of Notch ligand Jagged-1, subsequently activating Notch signaling in HSPCs and changing the differentiation potential of myeloid and lymphoid progenitor cells to induce AML (Kode et al., 2014). Ptpn11 is a positive regulator of the RAS signaling pathway in osteogenic progenitor cells. Activating mutation in Ptpn11 has been reported to overproduce chemokine CCL3 and recruit monocytes to the site of HSCs. Monocytes can produce inflammatory molecules to stimulate HSC differentiation and proliferation, causing juvenile chronic myelomonocytic leukemia (JMML) (Dong et al., 2016). The above researches proved that elimination or mutation of osteoblasts is the predisposing factor for hematological malignancies (Table 1).

**TABLE 1:** Contribution of hematopoietic and non-hematopoietic cells to hematopoietic homeostasis, hematologic malignancies, and after HSCT.

Cell population	Hematopoietic homeostasis		Hematologic malignancies		After HSCT
	Quiescence	Mobilization	Disease	Contribution or pathway	
Osteoblasts	Maintenance of early lymphoid		MDS AML	Deletion of Dicer1 (Raaijmakers et al., 2010)	–
	progenitors (Ding and Morrison, 2013)				
			AML	Mutation of β-catenin (Kode et al., 2014)	
	Hematopoietic regulation [TPO (Qian et al., 2007),		JMML	Mutation of Ptpn11 (Dong et al., 2016)	
	ANGPT1 (Arai et al., 2004) and				
	osteopontin (Nilsson et al., 2005)]				
			MPN	Functionally altered osteoblast (Schepers et al., 2013)	
Osteoclasts	Induction of osteoblasts development		MM	Protection of myeloma cells (PD-L1, CD38) (An et al., 2016)	–
Perivascular cells	NG2^+^ pericytes (Kunisaki et al., 2013)	Lepr^+^ pericytes (Kunisaki et al., 2013)	Leukemia	Inhibition of leukemia (reprogram macrophages) (Xia et al., 2020)	Recipient MSCs decreased and reconstruction of BM niche (Ding et al., 2014)
			MM	Promotion of MM growth (MSC-derived exosomes) (Roccaro et al., 2013)	
Endothelial cells	Regulation of HSPCs (Winkler et al., 2012) and		–	LSC engraftment (E-selectin and CXCL12) (Krause et al., 2014)	EPCs decreased and dysfunction (Kong et al., 2013; Cao et al., 2018)
	Maintenance of hematopoietic (Poulos et al., 2013)				
			AML	Chemoresistance to cytarabine (IL-8)	Reconstruction of hematopoietic function (Chute et al., 2007; Hooper et al., 2009; Himburg et al., 2016)
			AML	Inhibition of megakaryocytosis (IL-4) (Gao et al., 2019)	Reduction of GVHD (infuse EPCs) (Zeng et al., 2012)
Adipocytes	Negative regulators of HSC		AML	Energy for leukemia cells (Shafat et al., 2017)	–
	(Yokota et al., 2000; Naveiras et al., 2009)				
			AML	Remodeling the BM adipocytes (GDF15) (Yang et al., 2019)	
	Positive regulators of HSC (Zhou et al., 2017)		AML	Deficiency of erythropoiesis (Boyd et al., 2017)	
			–	Drug resistance of leukemia cells (Behan et al., 2009; Sheng et al., 2016, 2017)	
Regulatory T cells	Maintenance of B cell lymphopoiesis		–	Immune privilege for malignant cells (Wang et al., 2020)	Immune privilege for Allo-HSC (Hirata et al., 2018)
	(IL-7) (Pierini et al., 2017)				Prevention of GVHD (Mancusi et al., 2019; Riegel et al., 2020)
Macrophages	CD234/DARC (Hur et al., 2016)	CD169^+^ Macrophages (Chow et al., 2013)	AML	Polarization of macrophages to M2 phenotype (arginine enzyme) (Mussai et al., 2013)	–
	HSPC homing (ITGA4-dependent) (Li et al., 2018)		ALL	Polarization of macrophages to M2 phenotype (BMP4) (Valencia et al., 2019)	
			CLL	Polarization of macrophages to M2-phenotype (Galletti et al., 2016)	
Megakaryocytes	Secretion TGFβ and TPO		AML	Inhibition of MEP (IL-4) (Gao et al., 2019)	HSC engraftment (PDGF-BB) (Olson et al., 2013)
				Inhibition of HSPCs proliferation (Egr3) (Gong et al., 2018)	HSC expansion (Zhao et al., 2014)

**MDS, myelodysplastic syndromes; AML, acute myelocytic leukemia; CML, chronic myeloid leukemia; ALL, acute lymphoblastic leukemia; MPN, myeloproliferative neoplasm; JMML, juvenile chronic myelomonocytic leukemia; MM, multiple myeloma; LSC, leukemia stem cells; CSF1, colony-stimulating factor-1; SCF, stem cell factor; TGF-β,transforming growth factor-β; MSCs, mesenchymal stem cells; THPO, thrombopoietin; ANGPT1, angiopoietin1; IL-8, interleukin-8; Gfi1, growth factor-independent 1; GDF15, growth differentiation factor 15; BMP4, bone morphogenetic protein 4; –, not mentioned in the article or unreported.**

Osteoclasts play a crucial role in establishing the HSC niche. The HSC clonal expansion requires a physical area having a percentage of osteoblasts to osteoclasts of 25–75% (Christodoulou et al., 2020). Therefore, using traditional methods to characterize the HSC niche as an endosteal niche or vascular niche may be insufficient. One mechanism of multiple myeloma (MM) is the imbalance between bone formation and resorption. In MM, osteoclasts can directly inhibit proliferative CD4^+^ and CD8^+^ T cells by upregulating immune checkpoint molecules such as programmed death ligand 1 (PD-L1), CD38, and Galectin-9, thus establishing the immunosuppressive microenvironment to protect myeloma cells (An et al., 2016). MM relapse is associated with dormant myeloma cells. Evidence shows that the dormant state is reversible, and dormant myeloma cells can be reactivated by osteoclasts remodeling the endosteal niche (Lawson et al., 2015). Thus, reactivating dormant myeloma cells by promoting bone resorption and combining it with targeted therapies could help achieve MM long-term remission or even cure.

### Perivascular Cells

Perivascular cells, principally pericytes, have been identified in several human organs, they are tissue-specific precursors of MSCs (Yianni and Sharpe, 2019). The combined expression of signaling lymphocytic activation molecule family (Kiel et al., 2005), and new genetic markers, which including CTNAL1 (Acar et al., 2015) and HOXB5 (Chen et al., 2016), suggest that HSCs primarily exist in the perisinusoidal niches (Kiel et al., 2005; Acar et al., 2015; Chen et al., 2016). Interestingly, small arteries surrounded by rare NG2^+^ pericytes are associated with dormant HSCs, whereas sinusoid-associated leptin receptor^+^ (LepR^+^) cells are associated with less quiescent HSCs (Kunisaki et al., 2013). CXCL12-abundant reticular (CAR) cell subpopulations are differentially localized on the surface of sinusoids and arterioles and secrete specific cytokines to establish a perivascular niche (Baccin et al., 2020). CXCR4 is a specific receptor of stromal cell-derived factor 1 (SDF-1, also known as CXCL12), which functions in HSC homing (Lai et al., 2014). After HSCT, blocking the interaction of CXCR4/SDF-1 with the CXCR4 antagonist AMD3100 can recover hematopoietic function by inducing HSC proliferation (Green et al., 2016).

Mesenchymal stem cells (MSCs) are the precursor cells of BM stromal cells and have been widely used in treating immune-related diseases. In leukemia, MSC interact with leukemia cell. AML cells induced osteogenic differentiation in MSCs. Schepers et al. (2013) demonstrated that leukemic myeloid cells stimulated MSCs to overproduce functionally altered osteoblasts to form BM fibrosis in myeloproliferative neoplasia (MPN) and remodel the endosteal BM niche into a tumor microenvironment. Subsequent studies revealed that following co-culturing with leukemic cell lines, MSCs overexpress early stage osteoblast markers, including OSX and RUNX2 (Battula et al., 2017). Furthermore, mechanistic studies have identified that AML cells upregulated the expression of connective tissue growth factor (CTGF) in BM-MSCs, and activated Smad1/5 signaling, inducing BM-MSCs to differentiate into committed osteoprogenitors, but not mature osteoblasts (Hanoun et al., 2014; Battula et al., 2017). There is report has shown increased adipogenic potential of MSC in AML, decreasing expression of SOX9 and EGR2 increased adipogenic potential of AML-MSCs and enhanced their ability to support AML progenitor cells (Azadniv et al., 2020). Leukemia microenvironment reduces the proliferation and differentiation potential of MSCs. The development of NRAS mutant leukemia is accompanied by gradual functional degeneration of BM MSCs (Xia et al., 2020). *In situ* infusion of healthy donor-derived BM MSCs into the BM cavity can reprogram host macrophages to Arg1-positive phenotype with rapid tissue repair, thereby remodeling the leukemia BM microenvironment and inhibiting leukemia development (Xia et al., 2020). Moreover, impaired MSCs inhibit the generation of HPCs. As a negative regulator of osteoclast function, osteoprotegerin (OPG) can support the function of T-ALL MSCs and promote the proliferation of HPCs via the p38/ERK pathway (Lim et al., 2016). Exosomes are known to mediate intercellular communication. In MM, BM-MSC-derived exosomes with high levels of oncogenic proteins and cytokines are transferred to MM cells to promote MM tumor growth (Roccaro et al., 2013). These studies highlight the contribution of BM-MSCs to disease progression.

The conditioning regimen is a key step in HSC implantation to inactivate the recipient’s immune system before transplantation. Moreover, it destroys the BM microenvironment. Numerous studies have validated the function of MSCs in enhancing the efficiency of HSCT (Ball et al., 2007; Peng et al., 2015). According to studies, chemotherapy, used to induce remission, will not inhibit the function of MSCs; however, conditioning regimen can seriously compromise the proliferation characteristics of MSCs (Shipounova et al., 2017). For example, Ding et al. (2014) reported that the number of BM MSCs decreased sharply in the early stage after HSCT, the surviving MSCs supported hematopoiesis *in vitro* and inhibited lymphocyte proliferation. Surprisingly, donor-derived MSCs are not detected in the MSCs after HSCT (Ding et al., 2014), indicating that the recipient MSCs are mainly involved in reconstructing the BM microenvironment after HSCT.

### Endothelial Cells

BM endothelial cells (ECs) include arteriolar endothelial cells (AECs) and sinusoid endothelial cells (SECs). AECs secrete nearly all detectable endothelial-derived SCF in the BM (Xu et al., 2018), whereas SECs play indispensable roles in regulating HSC by secreting CXCL12 and a little SCF (Ding et al., 2012), and exclusively expressing adhesion molecule E-selectin (Winkler et al., 2012). Endothelial-specific angiocrine factor Jagged-1 can sustain hematopoietic homeostasis in a Notch-dependent manner by balancing the rate of self-renewal and differentiation of HSPCs (Poulos et al., 2013). In addition, endothelial cell-specific transcription factor Klf6 can directly regulate the expression of chemokine CCL25b and HSC expansion through CCL25b/CCR7 chemotactic signals (Xue et al., 2017).

**FIGURE 2:**
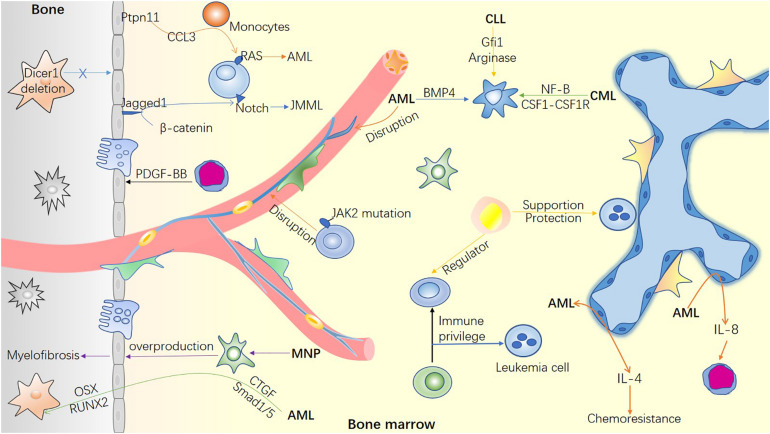
Bone marrow microenvironment in hematological malignancies. The mutation of microenvironment cells can induce hematological malignancies. Simultaneously, malignant cells invade and remold the blood microenvironment to facilitate their survival and inhibit the function of normal hematopoietic cells. The remodeled microenvironment can not only guide the homing of LSC, but also promote the immune escape of tumor cells and resistance to chemotherapy through various mechanisms.

ECs mediate leukemic stem cell (LSC) homing and engraftment. Sipkins et al. (2005) reported that leukemic cells preferably localize in vascular regions rich in E-selectin and CXCL12 where HSPCs home to. E-selectins and their ligands are required for LSC engraftment in the BM niche (Krause et al., 2014). Activated ECs are considered as potential mediators of leukemia relapse. Specifically, AML-induced activation of ECs stimulates interleukin-8 (IL-8) secretion, leading to a significant proliferation of non-adherent AML cells and chemoresistance to cytarabine (Vijay et al., 2019). Moreover, BM endothelial cells overproduce IL-4 to inhibit megakaryocytosis in AML. A combination of induction chemotherapy and inhibition of IL-4 not only recovers the platelet count but also prolongs the remission time in AML mice (Gao et al., 2019). These studies elucidate the link between endothelial cells and leukemia in the BM (Figure 2), thereby offering a potential therapeutic target in leukemia.

It was reported more than 25 years ago that damaged biomarkers of ECs, such as soluble thrombomodulin and von Willebrand factor (vWF), are increased significantly both before and after transplantation (Richard et al., 1996). Intercellular adhesion molecule-1, which represents activated ECs, was remarkably increased 3 weeks after transplantation (Richard et al., 1996). However, these markers have no diagnostic or prognostic value for complications related to transplants (Palomo et al., 2010). Subsequent studies have proved that circulating endothelial cells and endothelial progenitor cells (EPCs) can serve as endothelial damage markers during HSCT, with the potential to predict and diagnose several complications after HSCT (Almici et al., 2017; Ruggeri et al., 2018). A combined infusion of EPCs with HSCT can effectively repair BM micro-vessels, promote hematopoietic and immune reconstruction, reduce the occurrence of GVHD and other related complications (Zeng et al., 2012). After allogeneic hematopoietic stem cell transplantation (also HSCT), the number of EPCs decreased with dysfunction in patients with PGF and aGVHD, manifested as decreased migration and angiogenesis, and increased reactive oxygen levels and apoptosis. The damage degree of EPCs is positively correlated with the level of reactive oxygen species and apoptosis (Kong et al., 2013; Cao et al., 2018).

Hematopoietic recovery after lethal irradiation requires a complete vasculature. In traditional concepts, new blood vessels in the embryo will only be produced when the ECs divide. Plein et al. (2018) found that erythro-myeloid progenitors (EMPs) can differentiate into ECs, these ECs are recruited into pre-existing vasculature to repair damaged blood vessels and generate new blood vessels. This discovery will promote further research on using stem cells to repair damaged blood vessels after HSCT. The function of SECs is inhibited after HSCT, because they are highly sensitive to radiotherapy and chemotherapy. The regeneration of SECs, mediated by the VEGFR2 signaling pathway, is crucial for hematopoietic function reconstruction (Chute et al., 2007; Hooper et al., 2009; Himburg et al., 2016). In addition, AEC-derived SCF can promote HSC recovery after BM cell clearance (Xu et al., 2018). The Notch signaling pathway is involved in irradiation-induced BM injury, such that conditional disruption of Notch signaling increases the sensitivity to irradiation. Human Delta-like 1 (DLL1) fused with an RGD motif acts as an endothelium-targeted soluble Notch DLL1 ligand. As a new Notch activator, it can rescue severe myelosuppression caused by radiation injury and chemical drugs, and greatly promote hematopoiesis reconstruction after transplantation (Tian et al., 2016).

### Adipocytes

Previous studies have reported adipocytes as negative regulators of HSCs in the BM (Yokota et al., 2000; Naveiras et al., 2009). After chemotherapy or transplantation, the administration of peroxisome proliferator-activated receptor γ (PPAR-γ) antagonist, which inhibits adipogenesis, can enhance hematopoietic recovery (Naveiras et al., 2009; Zhu et al., 2013). However, recent studies have revealed that BM adipocytes (BMAs) positively regulate HSC regeneration under stress conditions. After radiotherapy or chemotherapy, physiological HSC nests are temporarily destroyed, MSCs initiate adipogenic differentiation urgently, and newly generated adipocytes form a temporary HSC niche to maintain the body’s basic hematopoietic function and promote HSC regeneration by secreting SCF. The “fat niche” is gradually replaced by physiological stem cell nests after BM reconstruction (Zhou et al., 2017). Recently, a study based on single-cell sequencing supported the above finding (Tikhonova et al., 2019). It was found that after 5-fluorouracil treatment, the expression of fat-related genes was upregulated in LepR + cell populations, whereas ontogenesis-related genes was downregulated (Tikhonova et al., 2019). Therefore, BMAs may have a dual role in HSC regulation.

BMAs release a series of inflammatory adipokines (leptin, TNF-α, and IL-6), as well as an anti-inflammatory adipokine (adiponectin) to regulate the proliferation and migration of leukemic cells (Beaulieu et al., 2011; Johrer et al., 2015; Ketterl et al., 2018). In contrast, leukemic blasts can program BMAs to support malignant cells. Leukemia cells can activate hormone-sensitive lipase to induce lipolysis. As an oxidative substrate, fatty acid-binding protein-4 carries fatty acids into the AML mitochondria and provides energy to leukemia cells (Shafat et al., 2017). It has been reported that AML cells differentiate the residual BMAs into small adipocytes by secreting growth differentiation factor 15 (GDF15). Moreover, transient receptor potential vanilloid 4 mediates GDF15-induced remodeling of BM adipocytes (Yang et al., 2019). A recent study revealed that in patients with AML during complete remission, chemotherapy drugs indirectly blocked MSC adipogenesis, enhanced the efficacy of consolidated chemotherapy, and prevented relapse by promoting GDF15 secretion from BM mononuclear cells (Liu et al., 2018). In addition, AML cells inhibited BMAs, resulting in erythropoiesis deficiency; this effect was reversed by the administration of PPAR-γ agonists *in vivo* (Boyd et al., 2017). In the end, BMAs can protect leukemic cells from drug- or radiation-induced oxidative stress (Sheng et al., 2016), prevent chemotherapy-induced apoptosis, increase the expression of pro-survival signals (Behan et al., 2009), absorb and metabolize chemotherapeutic drugs (Sheng et al., 2017), thus reducing the cytotoxicity of chemotherapeutic drugs and leading to cellular drug resistance of leukemia (Behan et al., 2009; Sheng et al., 2016, 2017; Figure 2). Targeting lipolysis may be an effective strategy for leukemia treatment and preventing drug resistance.

### Neural Regulation

Nerve fibers regulate HSCs through various signal transduction pathways (Figure 1). Early studies found that HSCs dynamically express catecholamine receptors (Spiegel et al., 2007). Sympathetic nerves transmit the adrenergic circadian rhythm signal, mediate the periodic release of HSPCs from BM to blood, by regulating the ability of BM stromal cells to secrete CXCL12 (Mendez-Ferrer et al., 2008). Osteoblasts express β2-adrenergic receptors and sympathetic nerves can inhibit osteoblast activity, it is necessary for G-CSF-induced HSPC mobilization (Asada et al., 2013). The loss of sympathetic nerves or adrenoreceptor β3 signaling is a potent driver of aging of the HSC niche, associated with the development of blood disorders (Maryanovich et al., 2018b). Evidence shows that neuropathy is associated with the progression of myeloproliferative neoplasms (MPNs). Nestin^+^ MSCs are in close contact with adrenergic nerves (Mendez-Ferrer et al., 2010), and the removal of sympathetic nerve fibers leads to reduced Nestin^+^ MSC cells, which in turn promotes HSC expansion and MPN development (Baryawno and Scadden, 2014). In addition, β3 adrenergic agonists restored the sympathetic regulation of Nestin^+^ MSC cells and prevented the progression of MPN. Remarkably, AML induces the sympathetic neuropathy at infiltrated sites, reduces the density of sympathetic nervous system network, expands Nes-GFP^+^ cells committed to differentiate to the osteoblast lineage, eventually promoting leukemia progression (Hanoun et al., 2014).

In addition to the catecholamine signals emitted by neurons, non-myelinated Schwann cells, which ensheathed the sympathetic nerves, can activate TGF-β produced by several cells in the microenvironment to maintain HSC quiescence (Yamazaki et al., 2011). Arranz et al. (2014) confirmed that HSCs with JAK2 mutation produced IL-1β to trigger BM nerve injury and Schwann cell death, whereas the administration of neuroprotective drugs or sympathomimetic drugs prevented the expansion of mutant HSCs. Furthermore, BM is innervated by parasympathetic nerves. Few studies have proved the role and mechanism of parasympathetic signals in regulating HSCs. Muscarinic receptor type-1 (Chrm1) is one of the acetylcholine receptors in the hypothalamus. Chrm1 signaling is from the central nervous system, initiated by glucocorticoid hormonal in the hypothalamus–pituitary–adrenal axis, can promote G-CSF-induced mobilization of HSCs (Pierce et al., 2017).

## Regulation by HSC Descendants

### Regulatory T Cells

Regulatory T cells (Tregs) play a vital role in building immune tolerance and preventing GVHD after allo HSCT. FoxP3^+^ Treg cells can maintain B cell lymphopoiesis by controlling the production of physiological IL-7 (Pierini et al., 2017). Niche-associated Tregs prevented malignant cells from immune attacks. Therefore, activated T cells were accumulated in the leukemic hematopoietic microenvironment, contributing to the progression of the disease (Wang et al., 2020). Tregs co-localize on the endosteal surface with allo HSCs after transplantation (Fujisaki et al., 2011), they can establish an immune-privileged “sanctuary” for donor-derived HSCs. Allo HSCs are rapidly lost after the depletion of FoxP3^+^ Treg cells (Fujisaki et al., 2011). Further, BM CD150^high^ Treg cells, located in the HSC niche, prevent allo HSC rejection, and facilitate allo HSC engraftment through adenosine (Hirata et al., 2018). Tregs are more susceptible to both intrinsic and extrinsic apoptotic pathways than the conventional T cells (Matsuoka et al., 2010; Weiss et al., 2011). Mitochondrial apoptotic priming of Tregs increased significantly in patients with mild and moderate chronic GVHD after HSCT (Murase et al., 2014). However, increased “priming” of all T-cell subsets reversed in patients with severe GVHD with typical lymphopenia, who received more intensive immunosuppressive therapy (Murase et al., 2014). Based on the characteristics of Tregs, adoptive Tregs transfer has prevented and treated GVHD (Mancusi et al., 2019; Riegel et al., 2020).

Two major subsets of T lymphocytes express CD4 and CD8 molecules, respectively, known as T-helper cell (Th) and T killer cell (Tc). Tc1 cells can produced perforin and granzyme, induced abnormal MK maturation, and destroyed PLT. Th1 and Th17 cells can secreted related cytokines to induce cytotoxic T cells and macrophage activation. The imbalance between T-helper cell 1 (Th1)/Th2, T killer cell 1 (Tc1)/Tc2, and Th17/Treg changes the level of cytokines and is associated with several complications. For example, Kong (Wang et al., 2016) found that as compared with good graft function (GGF) and healthy donors, the percentage of Th1 and Tc1 cells producing IFN-γ increased in patients with PGF after allo HSCT, whereas the percentage of Th2 and Tc2 cells that produce IL-4 decreased, resulting in an increased proportion of Th1/Th2 and Tc1/Tc2. This finding indicated that both CD4^+^ and CD8^+^ T cells were polarized toward type I immune response in patients with PGF. Another study found that the ratio of Th17/Treg in the BM of patients with PGF was significantly higher than in those with GGF (Kong et al., 2017). Therefore, an abnormal T cell response could contribute to the pathogenesis of PGF. Prolonged isolated thrombocytopenia (PT) is an independent risk factor for poor prognosis after allo HSCT (Kim et al., 2006). The proportion of Th1, Tc1, and Th17 cells increased significantly in patients with PT after allo HSCT (Song et al., 2017).

### Macrophages

Macrophages are derived from HSCs, they also participate in the formation of the hematopoietic microenvironment (Figure 1). In contrast, macrophages can directly regulate HSCs: They express CD234/DARC that interact with CD82 on the surface of LT-HSCs to inhibit cell cycle progression of LT-HSCs (Hur et al., 2016); CD169^+^ macrophages can promote erythroblast differentiate into reticulocytes and eliminate the aging erythrocyte (Chow et al., 2013); Similarly, VCAM-1^+^ macrophage-like niche cells interact with HSCs in an ITGA4-dependent manner and direct them homing to a specific niche (Li et al., 2018). The BM macrophages provide the HSC niche indirect support through osteoblasts and Nestin^+^ MSCs (Chow et al., 2011; Chang et al., 2014), Based on these results, we infer that depleting macrophages could induce HSC mobilization.

Leukemic cells create an immunosuppressive microenvironment for malignant cells by promoting the polarization of macrophages (Figure 2). For instance, in AML, the growth factor-independent 1 transcriptional repressor is involved in macrophage polarization (Al-Matary et al., 2016). Similarly, in AML, enzyme arginase enhances arginine metabolism, polarizes monocytes into M2 with immunosuppressive characteristics, and inhibits normal HSPC proliferation and differentiation (Mussai et al., 2013). In addition, interferon regulatory factor 7 promotes the polarization of tumor-related macrophages to M1 by activating the SAPK/JNK pathway. This in turn activates the IRF7-SAPK/JNK pathway to induce more M1-like macrophages and prolonging AML mice survival time (Yang et al., 2018). In acute lymphocytic leukemia (ALL) BM, the proportion of myeloid-derived suppressor cells and M2-like macrophages significantly increased (Hohtari et al., 2019). ALL cells induced the generation of immunosuppressive dendritic cells and M2-like macrophages by expressing bone morphogenetic protein 4 (BMP4) (Valencia et al., 2019). Macrophages in chronic lymphocytic leukemia (CLL) mice demonstrated an M2-like phenotype, and gene expression profiles revealed a high expression of programed death ligand-1 (PD-L1) (Hanna et al., 2016). It has been reported that CLL lymphocytes produce nicotinamide phosphoribosyl transferase after the NF-B signaling pathway is activated and induces M2 phenotype of macrophages (Audrito et al., 2015). Moreover, CLL could induce polarization of M2 macrophages via the colony-stimulating factor-1 (CSF1)-CSF1R pathway, and blocking CSF1R signal transduction can reduce the leukemia cell load (Galletti et al., 2016).

### Megakaryocytes

Megakaryocytes (MKs) constitute an integral part of the BM microenvironment (Table 1). The hematopoietic differentiation hierarchies are constantly updated, especially the origin of MKs. Adolfsson et al. (2005) proposed a new differentiation model, in which megakaryocyte/erythroid progenitor cells are derived directly from HSCs. In 2018, Frenette demonstrated that platelet and myeloid-based HSCs, marked by vWF expression, are regulated by MKs via CXCL4, whereas lymphoid-based vWF^–^ HSCs are located in and regulated by NG2 + arteriolar niches (Pinho et al., 2018). Furthermore, deleting MKs reprogram vWF^+^ HSCs from myeloid-based to a balanced lineage after transplantation (Pinho et al., 2018). The hematopoietic process of MKs is regulated by several cytokines and transcription factors, such as TPO and TGF-β. In the bone marrow of AML mice, MKs produced abundant TGFβ1; this overproduced and activated TGFβ1 directly upregulates Egr3 and inhibits the HSC cell cycle (Gong et al., 2018). After chemotherapy or radiotherapy, MKs can temporarily increase the secretion of fibroblast growth factor-1 and inhibit the TGF-β signal pathway, thus stimulating HSCs to enter the cell cycle and expand (Zhao et al., 2014). Thrombocytopenia is a common and fatal complication in patients with leukemia in clinic. In AML mouse, the number of megakaryocyte progenitor cells decreased sharply and the maturation of MKs was severely damaged too, it was surprising that overproduced IL-4 from bone marrow endothelial cells plays an inhibitory role in the differentiation of MKs (Gao et al., 2019). Host MKs play a novel role in promoting donor HSC implantation and expansion. After TBI, surviving MKs migrate to the endosteal surface of trabecular bone (Olson et al., 2013), secreting platelet-derived growth factor-BB to promote the proliferation of osteoblasts and significantly enhance donor HSC engraftment (Olson et al., 2013).

## Conclusion and Future Perspectives

The precise regulation of HSCs by the BM microenvironment is extremely complex. Further studies are required to check whether other niche cells and new factors exist in the BM. Although available literature focuses on how healthy BM microenvironment and leukemia microenvironment regulate HSCs, it lacks research on changes in the BM microenvironment before and after HSCT. In addition, targeted therapy of leukemia is a breakthrough in clinical treatment; however, problems such as drug resistance of targeted therapy, toxicity superposition of combined therapy, and lack of effective targets exist as well. In conclusion, there is a need for more novel therapeutic targets.

## Author Contributions

All authors drafted the manuscript, contributed to manuscript revision, and approved the final version of the manuscript.

## Conflict of Interest

The authors declare that the research was conducted in the absence of any commercial or financial relationships that could be construed as a potential conflict of interest.
